# Physical Restraint Use in Acute Care Hospitals: A Diagnostic Study on Knowledge, Documentation, and Patient Safety from a Humanization Perspective

**DOI:** 10.3390/healthcare14050694

**Published:** 2026-03-09

**Authors:** Alicia Albalat-Rodríguez, Ana Fernández-García, Violeta Hernández-De Arribas, Nuria Pérez-Panizo, Patricia Nieto-Alcantud, Sara Guillén-Tolbaños, Jesús De Cabo-Calvo, Marina De la Matta-Canto, Natalia Mudarra-García, Francisco Javier García-Sánchez

**Affiliations:** 1 Quality and Education Area, Hospital Universitario Ramón y Cajal, Instituto Ramón y Cajal de Investigación Sanitaria (IRYCIS), 28034 Madrid, Spain; alicia.albalat@salud.madrid.org (A.A.-R.); violeta.hernandez@salud.madrid.org (V.H.-D.A.); marinaalmu.matta@salud.madrid.org (M.D.l.M.-C.); 2 Psiquiatrics Hospitalization Area, Hospital Universitario Ramón y Cajal, Instituto Ramón y Cajal de Investigación Sanitaria (IRYCIS), 28034 Madrid, Spain; ana.fernandez.garcia@salud.madrid.org; 3 Geriatrics Service, Hospital Universitario Ramón y Cajal, Instituto Ramón y Cajal de Investigación Sanitaria (IRYCIS), 28034 Madrid, Spain; nuriamaria.perez@salud.madrid.org; 4 Surgical Hospitalization Area, Hospital Universitario Ramón y Cajal, Instituto Ramón y Cajal de Investigación Sanitaria (IRYCIS), 28034 Madrid, Spain; pnalcantud@salud.madrid.org; 5 Pulmonology Hospitalization Area, Hospital Universitario Ramón y Cajal, Instituto Ramón y Cajal de Investigación Sanitaria (IRYCIS), 28034 Madrid, Spain; sara.guillen@salud.madrid.org; 6 Internal Medicine Hospitalization Area, Hospital Universitario Ramón y Cajal, Instituto Ramón y Cajal de Investigación Sanitaria (IRYCIS), 28034 Madrid, Spain; jesus.cabo@salud.madrid.org; 7 Research Nursing Area, Hospital Universitario Ramón y Cajal, Instituto Ramón y Cajal de Investigación Sanitaria (IRYCIS), 28034 Madrid, Spain; 8 Department of Nursing, School of Medicine, Universidad San Pablo-CEU, CEU Universities, Urbanización Montepríncipe, 28660 Boadilla del Monte, Spain; 9 Emergency Room Service, Surgical Prehabilitation Unit, Hospital Universitario Infanta Cristina, Instituto de Investigación Sanitaria Hospital Puerta de Hierro Segovia Arana (IDIPHISA), 28981 Madrid, Spain; franci11@ucm.es; 10 Medical Department, Faculty of Medicine, University Complutense of Madrid, 28040 Madrid, Spain

**Keywords:** physical restraints, humanization care, nursing practice, hospital managements, healthcare quality

## Abstract

**Background:** The use of physical restraints in hospital settings remains a controversial practice due to its ethical, legal, and safety implications. Although restraints are intended to prevent falls or manage agitation, their inappropriate use may compromise patient dignity, autonomy, and quality of care. Current healthcare policies emphasize restraint reduction, appropriate documentation, and professional training as key elements of humanized and safe care. **Methods:** A descriptive cross-sectional study based on an anonymous self-administered survey was conducted in a tertiary university hospital as the diagnostic phase of a quality improvement project aimed at evaluating healthcare professionals’ knowledge, perceptions, and documentation practices related to physical restraint use. A structured ad hoc questionnaire was distributed to registered nurses and nursing assistants working in adult inpatient units using a non-probabilistic convenience sampling strategy. The survey explored training, clinical decision-making, communication with patients and families, awareness of institutional protocols, and use of the electronic health record (EHR). Descriptive analyses and Pearson’s chi-square tests were performed using IBM SPSS Statistics. **Results:** A total of 241 professionals participated. More than half of respondents (54.8%) reported no formal training in physical restraint use, and only 27.4% considered their training sufficient. Although 86.3% stated they were familiar with restraint indications, only 53.5% were aware of the existence of a structured EHR restraint registry, and just 31.0% consistently completed it. Documentation of restraint removal was particularly low (32.9%). Furthermore, significant discrepancies were observed between regulatory definitions of restraints and professionals’ perceptions regarding practices requiring formal documentation. Statistically significant associations were identified between professional category, perceived training adequacy, and knowledge of physical restraint indications. **Conclusions:** This diagnostic phase identified substantial gaps between regulatory requirements, professional knowledge, and real-world documentation practices related to physical restraint use. The findings highlight the need for competency-based training strategies, standardized documentation processes, and strengthened institutional leadership to promote patient safety, regulatory compliance, and the humanization of hospital care.

## 1. Introduction

The use of physical restraints in hospital settings has become an increasingly debated issue in recent years, particularly within the framework of patient safety, humanized care, and respect for fundamental rights. Physical restraints are commonly defined as any method, device, or action that limits an individual’s freedom of movement and that the person cannot easily remove or control. Although traditionally applied to prevent falls, manage agitation, or avoid accidental removal of medical devices, their use must be considered an exceptional measure rather than a routine intervention [1].

Scientific evidence has consistently shown that the use of physical restraints is associated with significant adverse outcomes, including physical injuries, psychological distress, functional decline, and deterioration of the therapeutic relationship between patients and healthcare professionals. These consequences are especially relevant in vulnerable populations, such as older adults, patients with cognitive impairment, and those experiencing acute confusion or delirium. Moreover, the use of restraints has not been shown to effectively reduce adverse events such as falls; in some cases, it may even increase their incidence [2].

In response to these concerns, international and national organizations have promoted strategies aimed at reducing restraint use and encouraging safer, less invasive alternatives. The World Health Organization has emphasized that restraint practices raise important ethical and human rights issues and should be minimized through preventive approaches, staff training, and organizational commitment. Similarly, healthcare policies in Spain and other European countries have increasingly incorporated restraint reduction as a key indicator of quality, safety, and humanization of care [3,4].

Within the Spanish healthcare system, the regulatory framework governing physical restraint use establishes strict requirements regarding medical prescription, proportionality, time limitation, periodic reassessment, patient and family information, and systematic documentation in the electronic health record. In the Community of Madrid, Resolution 106/2017 of the Regional Health Authority explicitly defines physical restraints, outlines professional responsibilities, and mandates structured documentation to ensure traceability and accountability [5]. Despite these regulations, previous studies suggest that compliance with documentation and reassessment requirements remains inconsistent in routine clinical practice.

One of the main challenges in reducing inappropriate restraint use is the gap between regulatory standards, professional knowledge, and real-world clinical practice. Healthcare professionals may perceive restraints as necessary safety measures, particularly in contexts of high workload, staffing constraints, or limited access to preventive alternatives. In this context, insufficient training, lack of awareness of institutional protocols, and poorly designed documentation systems can contribute to the normalization of restraint use and to under-registration in electronic health records.

Training and education have been identified as central components in strategies to reduce restraint use and improve compliance with ethical and legal standards. Multicomponent interventions that combine staff education, protocol implementation, audit and feedback, and leadership involvement have demonstrated reductions in restraint prevalence and improvements in documentation quality [2,6]. However, the effectiveness of such interventions largely depends on an accurate understanding of baseline knowledge, perceptions, and practices among healthcare professionals.

From a humanization perspective, the appropriate management of physical restraints is closely linked to patient-centered care, shared decision-making, and respect for dignity and autonomy. Transparent communication with patients and families, along with systematic documentation of indication, duration, reassessment, and withdrawal, is essential to ensure that restraint use, when unavoidable, is ethically justified and clinically appropriate [7,8].

In this context, the present study represents the diagnostic phase of a multi-phase quality improvement project conducted in a tertiary university hospital. The aim of this initial phase was to assess healthcare professionals’ knowledge, perceptions, and documentation practices related to physical restraint use in adult inpatient units. By identifying gaps in training, conceptual understanding, and electronic health record use, this study seeks to provide the foundation for the subsequent implementation of a structured training program and a standardized documentation circuit, aligned with patient safety principles, regulatory compliance, and the humanization of hospital care.

Despite the growing ethical and regulatory attention devoted to physical restraint use, significant variability persists in clinical practice, professional perceptions, and documentation processes across hospital settings. Current evidence mainly focuses on patient outcomes or intervention programs, while limited attention has been paid to organizational and professional factors influencing restraint decision-making and registration practices. Previous studies have shown that healthcare professionals’ attitudes, beliefs, and previous experiences play a decisive role in restraint use, contributing to significant variability between institutions and even among professionals working within the same clinical setting [9,10,11]. International evidence indicates that restraint practices in acute hospitals are frequently influenced by organizational culture, implicit routines, and interprofessional dynamics rather than standardized clinical decision-making processes [2,12,13]. Furthermore, healthcare staff perceptions and training levels have been identified as key determinants in both the initiation and maintenance of physical restraints, highlighting the need to better understand contextual and professional factors influencing their use in hospital environments [9,10,11]. Therefore, a diagnostic assessment of baseline knowledge, perceptions, and institutional practices is required before implementing structured improvement strategies. This study represents the first phase of a broader quality improvement project aimed at identifying gaps between regulatory recommendations and real-world clinical practice.

This study represents the diagnostic phase of a broader institutional quality improvement strategy aimed at optimizing physical restraint use, documentation practices, and professional training within hospital care settings.

## 2. Materials and Methods

### 2.1. Study Design

A descriptive, observational, cross-sectional study was conducted as the diagnostic phase of a multi-phase interventional project aimed at improving the prescription, documentation, and management of physical restraints in hospital settings. This initial phase focused on assessing healthcare professionals’ knowledge, perceptions, and documentation practices prior to the implementation of a structured training and quality improvement strategy.

### 2.2. Setting

The study was carried out at a tertiary-level university hospital in Madrid, Spain. Data collection took place in adult inpatient units, including medical and surgical wards.

Physical restraint episodes must be documented in the institutional electronic health record (HCIS), which includes mandatory fields related to indication, medical prescription, reassessment, and removal. Completion of this registry constitutes an institutional requirement linked to patient safety monitoring and quality assurance processes.

### 2.3. Participants

The target population consisted of nursing professionals, including registered nurses (RN) and nursing assistants (TCAE), working in adult inpatient units.

A non-probabilistic convenience sampling strategy was used to recruit nursing professionals (registered nurses and nursing assistants) working in adult inpatient units of the hospital. Participation was voluntary and anonymous.

The questionnaire was distributed both in person and through an institutional electronic link provided to unit supervisors for internal dissemination. During the data collection period, no individual reminder invitations were sent to potential participants. However, unit supervisors issued a single additional informational communication within their teams, without nominal identification, to remind staff of the availability of the survey.

The survey was distributed to a total of 277 eligible professionals (registered nurses and nursing assistants) working in adult inpatient units. Of these, 241 completed the questionnaire, resulting in a response rate of 87.0%. Approximately 15% of the total potential participants were not included in the final sample: 10% corresponded to eligible professionals who did not respond, and 5% corresponded to professionals with temporary contracts who did not meet the inclusion criteria.

#### 2.3.1. Inclusion Criteria

- Registered nurses and nursing assistants employed in adult inpatient wards.
- Permanent, temporary, or interim staff members.

#### 2.3.2. Exclusion Criteria

- Healthcare professionals in training (students, residents, or trainees) have limited involvement in clinical decision-making regarding the use of physical restraints, and their knowledge of institutional procedures may not be representative.
- Staff assigned to temporary rotations (short-term replacements or occasional support personnel) may have insufficient or sporadic exposure to unit protocols and participation in structured documentation processes, potentially introducing unwanted variability into the results.

### 2.4. Questionnaire Development

An ad hoc questionnaire was developed based on institutional clinical practice needs and existing regulatory requirements related to physical restraint use and documentation. The instrument was designed according to the institutional restraint protocol and the electronic health record registry (HCIS). Content review was conducted by members of the hospital Quality and Patient Safety Committee to ensure clarity and clinical relevance.

The questionnaire was distributed electronically using Microsoft Forms through institutional communication channels and accessed voluntarily via QR code. Participation was anonymous.

As this study corresponds to a diagnostic phase of a quality improvement initiative, no psychometric validation or reliability analysis of the instrument was performed. This limitation is acknowledged and discussed accordingly.

Although validated instruments such as the Perception of Restraint Use Questionnaire (PRUQ) have demonstrated adequate psychometric properties for assessing professionals’ perceptions toward restraint use, these tools primarily explore attitudinal dimensions rather than compliance with local regulatory and documentation requirements. Therefore, an ad hoc questionnaire was developed to specifically assess knowledge and practices related to institutional protocols and regional legal frameworks governing physical restraint use within the study setting.

### 2.5. Data Collection Instrument

Data were collected using an anonymous, self-administered structured questionnaire (version V5) developed ad hoc by the research team and implemented using Microsoft Forms. Access to the survey was provided through a QR code distributed across the participating units.

The questionnaire consisted of 34 items grouped into eight sections:

1. Sociodemographic and professional characteristics.
2. Knowledge and perceptions regarding physical restraints.
3. Prescription practices and clinical decision-making.
4. Communication with patients and families.
5. Scope and indications for restraint use.
6. Documentation practices in the electronic health record (HCIS).
7. Training and educational resources.
8. Legal framework and professional responsibility.

### 2.6. Variables

The following variables were collected:

- Sociodemographic and professional variables: sex, age, professional category, clinical unit, years of professional experience, and employment status.
- Training-related variables: previous training in physical restraints and perceived adequacy of training.
- Knowledge and perception variables: understanding of restraint indications, perceived appropriateness of restraint use, and awareness of institutional protocols.
- Clinical practice variables: physician involvement in prescription and withdrawal of restraints, communication with patients and families, and reassessment frequency.
- Documentation variables: awareness and use of the structured restraint registry in the electronic health record (HCIS), completion of initiation and termination records.
- Legal and organizational variables: knowledge of the legal framework and perceived need for improved documentation tools.

### 2.7. Ethical Considerations

The study complied with current data protection regulations, including Regulation (EU) 2016/679 (General Data Protection Regulation) and Spanish data protection legislation (Organic Law 3/2018). The study was conducted in accordance with the ethical principles of the Declaration of Helsinki and was approved by the Nursing Directorate, and subsequently submitted to the hospital’s Nursing Research Department (2025/005, 04/07/2025). Participation was voluntary and anonymous, and no personally identifiable information from professionals was collected.

Physical restraint use in the study institution is regulated according to regional healthcare legislation and institutional protocols. Restraints require a medical prescription following patient assessment and must be documented in the electronic health record (HCIS). In exceptional situations involving immediate risk to patient safety, nursing professionals may initiate temporary restraint measures, which must subsequently be medically validated and formally prescribed. All procedures follow the institutional protocol derived from Resolution 106/2017 of the Madrid Regional Health Service, ensuring proportionality, time limitation, and periodic reassessment.

### 2.8. Statistical Analysis

Statistical analyses were performed using IBM SPSS Statistics for Windows, Version 29.0 (IBM Corp., Armonk, NY, USA). Descriptive statistics were calculated using absolute and relative frequencies for categorical variables. Associations between selected Categorical variables were analyzed using Pearson’s chi-square test to assess associations between study variables. When expected cell frequencies were lower than five in more than 20% of contingency table cells, Fisher’s exact test was applied as appropriate.

For all comparisons, contingency tables were constructed, and chi-square $\chi^{2}$ or Fisher’s exact test statistics were calculated, along with degrees of freedom (when applicable) and corresponding *p*-values. Statistical significance was established at *p* < 0.05.

Specifically, the following associations were evaluated

1. The association between knowledge of physical restraint indications (yes/no) and variables related to training, perception, clinical practice, and documentation.
2. The association between the perception of having received sufficient training (yes/no) and variables related to knowledge, clinical practice, and documentation.
3. The association between professional category (registered nurse vs. nursing assistant) and variables related to training, knowledge, restraint use practices, and documentation.

For all comparisons, contingency tables were analyzed, and chi-square ($\chi^{2}$) values, degrees of freedom (df), and *p*-values were calculated. Statistical significance was established at *p* < 0.05.

## 3. Results

### 3.1. Participant Characteristics

A total of 241 healthcare professionals participated in the study, all of whom worked in adult inpatient units. The sample was predominantly female (88.4%), with registered nurses representing 61.0% of participants and nursing assistants (TCAE) accounting for 39.0%. Participant characteristics are summarized in Table 1.

Most professionals (83.0%) had less than 10 years of service. Participation was obtained from a wide range of clinical units, with the highest representation from Internal Medicine (12.0%), Cardiology (8.7%), Psychiatry (8.3%), General and Digestive Surgery (7.9%), Infectious Diseases (7.9%), Pulmonology (7.5%), and Orthopedics/Orthogeriatrics (7.5%).

### 3.2. Training and Perception of Physical Restraint Use

More than half of participants (54.8%) reported having received no specific training in physical restraints, and only 27.4% considered their training sufficient. Despite this, 66.4% perceived the use of physical restraints as appropriate in the hospital setting. Training and perceptions regarding physical restraint use are summarized in Table 2, while additional association analyses are provided in Supplementary Table S1.

An overwhelming majority of respondents (96.3%) expressed a need for additional training focused on restraint prevention, and 72.6% believed that the use of restraints was directly influenced by the level of training received, as shown in Table 3. Comparative analyses between psychiatry units and other units are provided in Supplementary Table S2.

### 3.3. Knowledge, Prescription Practices, and Communication

Although 86.3% of participants reported knowing the clinical indications for the use of physical restraints, inconsistencies were observed in prescription and withdrawal practices. Only 19.9% stated that physicians always assessed patients prior to restraint prescription, and just 17.4% reported that restraint removal was always prescribed by a physician. Details regarding prescription practices and communication are presented in Table 4. Associations between knowledge of restraint indications and professional characteristics are presented in Table 5 and Supplementary Table S4.

Communication with patients and families was irregular; only 19.9% indicated that patients and relatives were always informed before restraint application, while the most frequent response was “sometimes” (30.3%), see Table 4.

### 3.4. Conceptualization of Physical Restraints and Documentation Criteria

Participants demonstrated heterogeneous interpretations of what constitutes a physical restraint. While combined immobilization systems (e.g., bed rails plus belts and wrist restraints) were commonly identified as restraints, lower-intensity measures such as raised bed rails alone were frequently excluded from this classification.

Similarly, documentation practices reflected a tendency to register only what professionals perceived as “maximum immobilization,” leading to the under-registration of lower-intensity restraint practices despite their inclusion in regulatory definitions.

### 3.5. Reassessment Practices

Regarding the frequency of reassessment, 40.9% of participants considered that restraints should be reassessed every 2 h, while 24.5% believed it should be done every 30 min. These frequencies align with patient safety standards; however, this awareness was not consistently translated into documented clinical practice. Perceived reassessment frequencies are summarized in Table 6.

### 3.6. Knowledge and Use of the Electronic Health Record Registry

Only 53.5% of respondents were aware of the existence of a structured physical restraint registry in the electronic health record (HCIS). Among those who were aware of the registry, only 31.0% consistently completed the documentation when restraints were applied.

Documentation of restraint termination was even lower, with only 32.9% reporting consistent completion. Nearly half of participants (49.4%) considered that an additional or clearer documentation tool was necessary to improve registration practices. Factors associated with the perceived need for an additional electronic registry tool are detailed in Supplementary Table S3.

Knowledge and use of the electronic health record registry are detailed in Table 7.

### 3.7. Training Resources

Engagement with existing institutional training resources was limited. Only 10.8% of professionals reported having viewed training modules on restraint use, 9.1% on restraint prevention, and 7.5% on the legal framework, indicating low penetration of current educational initiatives. Engagement with institutional training resources is shown in Table 8.

### 3.8. Institutional Audit Findings

In 2024, a total of 83 physical restraint episodes were recorded in the electronic health record system. However, during the same period, the acquisition of restraint devices reached 10,494 units, including wrist restraints and abdominal belts.

The difference observed between the volume of acquired devices and the number of documented records reveals a relevant discrepancy between the potential use of physical restraints and their formal registration within the electronic health record system. This divergence supports the existence of structural under-reporting of physical restraint use in routine clinical practice.

## 4. Discussion

The results of this study confirm the continued use of physical restraints in hospital settings and reveal significant deficiencies in knowledge, documentation, and compliance with regulatory requirements among healthcare professionals. These findings are consistent with concerns raised in previous studies and institutional reports highlighting the gap between policy recommendations, clinical practice, and documentation in the management of physical restraints [2,5,6,14].

From an organizational perspective, these findings may be interpreted using the Donabedian framework, in which structural components such as institutional protocols and training strategies interact with process-related factors, including professional decision-making and documentation practices. The discrepancy observed between regulatory requirements and actual clinical documentation suggests weaknesses at the process level that may compromise patient safety monitoring and quality assessment.

### 4.1. Deficit in Documentation and Traceability

One of the most relevant findings of this diagnostic phase is the low level of completion of the structured physical restraint registry in the electronic health record (HCIS). Although more than half of the professionals were aware of the existence of the registry, only a minority reported completing it consistently, and documentation of restraint removal was particularly limited. This finding is especially relevant from a patient safety and legal perspective, as proper documentation is essential to ensure traceability, accountability, and compliance with current regulations [5].

The institutional audit findings further reinforce this interpretation, revealing a marked discrepancy between the absence of registered restraint episodes in the electronic health record and the documented consumption of restraint devices during the same period. This mismatch supports the hypothesis of systematic under-reporting rather than absence of restraint use, highlighting documentation practices as a critical patient safety concern.

**Figure 1:**
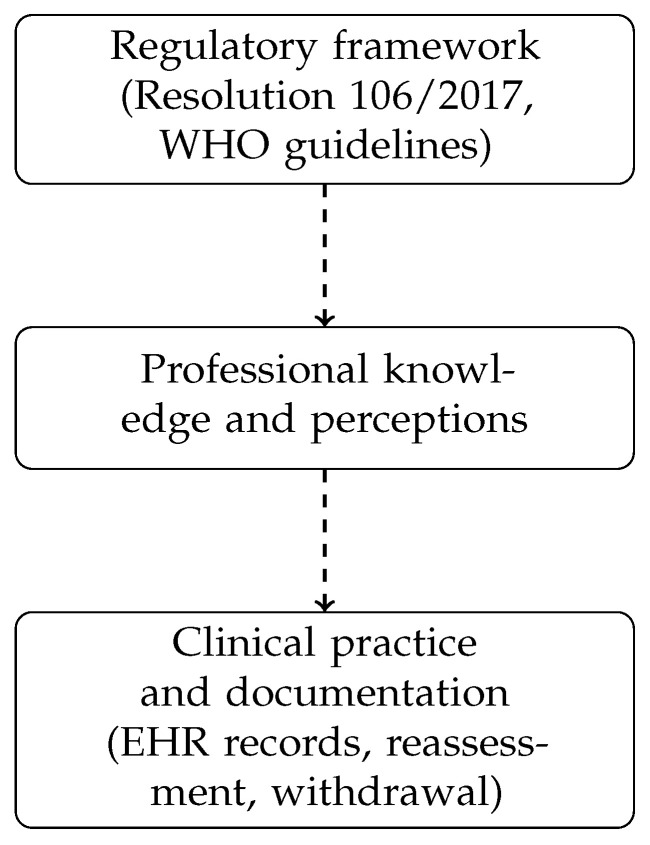
Conceptual model illustrating the gap between regulatory requirements, healthcare professionals’ knowledge, and real-world clinical practice in physical restraint use.

The discrepancy observed between the number of registered restraints and the consumption of restraint devices detected through material management records and institutional audits reinforces the presence of under-registration in routine clinical practice. Similar situations have been described in the literature, where the absence of standardized and user-friendly documentation systems has been identified as a major barrier to the appropriate monitoring and reduction of physical restraint use in hospitals [3,4] (Figure 1).

These results suggest that isolated educational interventions alone may be insufficient to modify clinical practice. Short training modules or informational resources, although necessary, may not lead to sustained behavioral change without organizational reinforcement, leadership involvement, and system-level monitoring strategies.

### 4.2. Knowledge and Training of Healthcare Professionals

Although most participants reported knowing the indications for the use of physical restraints, more than half had not received specific training on this topic, and only a small proportion considered their training sufficient. This lack of formal education may partly explain the inconsistencies observed in prescription practices, reassessment, and documentation. Previous studies have highlighted that insufficient training contributes to the normalization of physical restraints as routine safety measures rather than exceptional interventions [15,16,17].

Evidence suggests that educational interventions alone are insufficient unless supported by organizational strategies, leadership engagement, and structured implementation frameworks [14].

The high percentage of professionals expressing the need for additional training, particularly focused on prevention, supports the relevance of the educational component proposed in the subsequent phases of this project. Furthermore, the fact that most respondents considered that restraint use depends on the training received suggests that educational interventions may have a direct impact on clinical practice, as previously reported in national and international studies [16,17].

This finding may reflect a process of cultural normalization of restraint use within hospital environments, where perceived patient safety risks, workload pressures, and organizational routines influence clinical decision-making beyond formal regulatory recommendations.

### 4.3. Prescription, Reassessment, and Professional Responsibility

The results reveal notable variability in prescription and reassessment practices. Only a minority of professionals indicated that physical restraints were always prescribed and withdrawn following medical assessment, and reevaluation frequencies were inconsistently applied. These findings indicate partial non-compliance with the regulatory framework, which establishes that restraint use must be medically prescribed, time-limited, and subject to periodic reassessment [5].

From a clinical governance perspective, the lack of systematic medical involvement in prescription and withdrawal represents a potential risk, as it places a disproportionate burden of responsibility on nursing staff. This issue has also been identified by professional societies, which emphasize the shared responsibility of physicians and nurses in the decision-making and monitoring process [12].

Variability in restraint practices has been consistently reported across acute care settings and is strongly associated with professional attitudes, institutional culture, and the availability of clinical guidance [9,11].

These findings highlight the shared responsibility between physicians and nursing professionals in restraint decision-making processes, particularly in situations requiring immediate patient safety interventions.

### 4.4. Communication with Patients and Families

Communication with patients and families regarding the use of physical restraints was found to be irregular. Only a small proportion of professionals reported always informing patients and relatives before applying restraints, while the most frequent response indicated that information was provided only occasionally. This finding is particularly relevant in the context of humanized care, as transparent communication is essential to respect patient autonomy and maintain trust in the therapeutic relationship [7,8].

The lack of systematic information provision suggests that communication is not yet fully integrated into routine restraint management. Incorporating explicit documentation fields related to patient and family information in the electronic health record may contribute to improving transparency and compliance with ethical standards.

### 4.5. Conceptualization of Physical Restraints

The study also identified heterogeneity in professionals’ perceptions of what constitutes a physical restraint. While high-intensity immobilization measures were widely recognized, lower-intensity practices such as bed rails or partial containment techniques were often excluded from this definition. This finding is consistent with previous reports describing conceptual ambiguity among healthcare professionals and highlights the need for clearer definitions and practical examples during training [18].

This conceptual discrepancy has direct implications for documentation, as professionals tend to register only measures perceived as more restrictive, leading to the under-registration of other practices that are nonetheless included in the regulatory definition of physical restraints [5].

**Figure 2:**
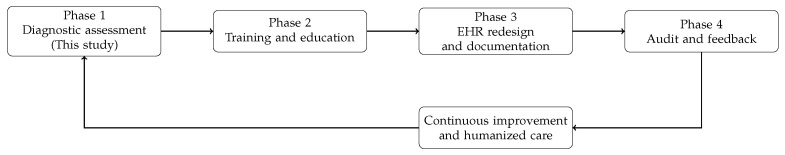
Multiphase quality improvement strategy for optimizing physical restraint use, documentation, and humanized care in hospital settings.

In this regard, the multiphase quality improvement strategy proposed in this project (Figure 2) aligns with international recommendations and national policies on patient safety and humanization of care.

This normalization of restraint use has been described in previous studies, where restrictive practices become embedded within routine care despite ethical concerns and limited evidence supporting their effectiveness [12,19].

### 4.6. Implications for Practice and Quality Improvement

Taken together, the results of this diagnostic phase highlight the need to implement a structured and coordinated strategy aimed at improving the management of physical restraints in hospital settings. This strategy should include targeted training programs, the redesign of documentation circuits in the electronic health record, and regular audits to monitor compliance and progress. Evidence from previous studies suggests that combining these elements is more effective than isolated interventions [2,14].

The findings of this study provide a solid baseline for the implementation of the subsequent phases of the project, which will evaluate the impact of educational and organizational interventions on documentation quality, professional practice, and institutional monitoring of physical restraint use.

Taken together, these findings indicate that physical restraint use should not be understood solely as an individual clinical decision but as an organizational phenomenon influenced by institutional culture, documentation systems, and professional training structures.

From an ethical perspective, the use of restraints represents a form of coercive practice that challenges patient autonomy and dignity, reinforcing the need for restraint-free care models aligned with human rights-based healthcare approaches [13].

## 5. Limitations

This study presents several limitations that should be considered when interpreting the results. First, the data were based on self-reported perceptions, which may not fully reflect actual clinical practice and could introduce social desirability bias, particularly in issues related to physical restraint use and compliance with documentation procedures.

Second, a non-probabilistic convenience sampling strategy was used, which may limit sample representativeness and affect the external validity of the findings. Additionally, the single-center design may restrict the generalizability of the results to other healthcare settings with different organizational, educational, or cultural characteristics.

Another limitation relates to the use of a self-administered questionnaire which, although ensuring anonymity and facilitating participation, may introduce variability in item interpretation and response accuracy. Furthermore, it was not possible to directly contrast professionals’ perceptions with objective data extracted from the electronic health record; therefore, discrepancies may exist between reported practices and actual documented clinical activity.

In addition, the questionnaire used in this study was specifically developed for institutional diagnostic purposes and did not undergo formal psychometric validation or reliability testing. Consequently, the results should be interpreted as exploratory and context-specific rather than as measurements obtained using a standardized validated instrument.

Finally, the cross-sectional design of the study prevents establishing causal relationships between training, professional perceptions, and restraint use practices. The findings should therefore be interpreted as associative rather than causal.

Despite these limitations, the primary aim of the study was to identify local gaps in knowledge, perceptions, and documentation practices in order to guide a context-adapted quality improvement intervention, rather than to extrapolate the findings to other hospital settings.

## 6. Conclusions

This diagnostic study highlights the existence of relevant gaps in the management of physical restraints in adult inpatient units of a tertiary hospital, particularly in relation to professional training, documentation practices, and compliance with the regulatory framework. Although most healthcare professionals report knowing the general indications for physical restraint use, this knowledge is not consistently reflected in clinical practice or in systematic documentation within the electronic health record.

The results show a low level of completion of the structured restraint registry, especially regarding restraint withdrawal and reassessment, as well as inconsistencies in medical prescription and communication with patients and families. In addition, notable discrepancies were identified between regulatory definitions of physical restraints and professionals’ perceptions of which practices require registration, contributing to under-documentation and limited traceability.

The high proportion of professionals reporting insufficient training and expressing a clear demand for additional education, particularly focused on prevention, reinforces the need to strengthen structured and standardized training strategies. These findings support the implementation of an integrated approach combining professional education, redesign of electronic documentation circuits, and regular auditing processes to improve compliance, patient safety, and accountability.

Overall, this diagnostic phase provides a solid baseline for the subsequent phases of the project, aimed at evaluating the impact of targeted educational and organizational interventions. Improving the management of physical restraints is essential to advance toward safer, more transparent, and more humanized hospital care, in line with current regulatory requirements and quality standards.

## 7. Future Directions

Future phases of the project will evaluate the impact of the implemented training program and the redesigned documentation circuit on actual restraint use, documentation quality, and audit outcomes. Longitudinal analyses will allow the assessment of whether improvements in knowledge and perception translate into sustained changes in clinical practice.

Future strategies should integrate organizational change, interdisciplinary responsibility, and competency-based training programs to move toward restraint-free hospital care.

## Figures and Tables

**Table 1:** Sociodemographic and professional characteristics of participants (n = 241).

Variable	Category	n	%
Sex	Female	213	88.4
	Male	27	11.2
	Non-binary	1	0.4
Age group (years)	<20	3	1.2
	20–30	44	18.3
	31–40	66	27.4
	41–50	53	22.0
	51–60	55	22.8
	>60	20	8.3
Professional category	Registered nurse	147	61.0
	Nursing assistant (TCAE)	94	39.0
Years of service	0–10	200	83.0
	11–20	22	9.1
	21–30	11	4.6
	>31	8	3.3

**Table 2:** Training and perception regarding physical restraint use (n = 241).

Variable	Response	n	%
Received prior training	Yes	109	45.2
	No	132	54.8
Perceives restraint use as appropriate	Yes	160	66.4
	No	81	33.6
Considers training sufficient	Yes	66	27.4
	No	175	72.6
Needs additional training	Yes	232	96.3
	No	9	3.7
Believes restraint use depends on training	Yes	175	72.6
	No	66	27.4

**Table 3:** Association between perceived adequacy of training in physical restraint management and professional, training, and documentation variable.

Variable	Training Sufficient n (%)	Training Not Sufficient n (%)	*p*-Value
Professional category (Nurse)	29 (43.9)	118 (67.4)	<0.001
Previous psychiatry experience	39 (59.1)	57 (32.6)	<0.001
Received training in restraints	52 (78.8)	57 (32.6)	<0.001
Restraint use considered appropriate	52 (78.8)	108 (61.7)	0.012
Knowledge of immobilization protocol	39 (59.1)	37 (21.1)	<0.001
Awareness of HCIS restraint registry	47 (71.2)	82 (46.9)	<0.001
Knowledge of registry location in HCIS	42 (63.6)	53 (30.3)	0.002
Completion of HCIS registry	24 (36.4)	16 (9.1)	<0.001
Completion of restraint removal documentation	17 (25.8)	8 (4.6)	0.042
Viewed restraint-related training module	13 (19.7)	13 (7.4)	0.006
Need for additional restraint training	59 (89.4)	173 (98.9)	<0.001
Knowledge of legal framework	44 (66.7)	38 (21.7)	<0.001
Knowledge of restraint indications	66 (100)	142 (81.1)	<0.001

Abbreviations: HCIS, Healthcare Information System.

**Table 4:** Prescription practices and communication with patients and families (n = 241).

Variable	Response	n	%
Knows restraint indications	Yes	208	86.3
	No	33	13.7
Physician assesses before prescription	Always	48	19.9
	Almost always	65	27.0
	Sometimes	64	26.5
	Almost never	57	23.7
	Never	2	0.8
	Unknown	5	2.1
Physician prescribes removal	Always	42	17.4
	Almost always	33	13.7
	Sometimes	51	21.2
	Almost never	73	30.3
	Never	28	11.6
	Unknown	14	5.8
Patients/families informed	Always	48	19.9
	Almost always	63	26.1
	Sometimes	73	30.3
	Rarely/Never	53	22.0
	Unknown	4	1.7

**Table 5:** Association between knowledge of physical restraint indications and training, perception, and documentation practices.

Variable	Knowledge Present n (%)	Knowledge Absent n (%)	*p*-Value
Received training in physical restraints	107 (51.4)	2 (6.1)	<0.001
Restraint use considered appropriate	145 (69.7)	15 (45.5)	0.006
Knowledge of institutional protocol	73 (35.1)	3 (9.1)	0.003
Correct identification of preventive strategy	95 (46.3)	12 (37.5)	0.350
Correct identification of physical restraint	54 (26.3)	6 (18.8)	0.358
Awareness of HCIS restraint registry	116 (55.8)	13 (39.4)	0.080
Completion of HCIS registry	38 (18.3)	2 (6.1)	0.071
Documentation of restraint removal	24 (11.5)	1 (3.0)	0.015
Perceived sufficient training	66 (31.7)	0	<0.001
Knowledge of legal framework	81 (38.9)	1 (3.0)	<0.001

Abbreviations: HCIS, Healthcare Information System.

**Table 6:** Perceived reassessment frequency of physical restraints (n = 241).

Reassessment Frequency	n	%
Every 30 min	58	24.5
Every 2 h	97	40.9
Every 4 h	31	13.1
Every 8 h	24	10.1
More than 8 h	13	5.5
Every 30 min		
and every 2 h	7	3
Every 2 and 4 h	4	1.7
Every 4 and 8 h	2	0.8
Every 8 h and		
more than 8 h	1	0.4

4 answers missed. Right answer: Every 30 min and every 2 h.

**Table 7:** Knowledge and use of the electronic health record registry for physical restraints (n = 241).

Variable	Response	n	%
Aware of registry existence	Yes	129	53.5
	No	112	46.5
Completes registry when applied	Always	40	31.0
(from aware of registry existence group	Sometimes	36	27.9
n = 129)	No	43	33.3
	Never applied restraints	10	7.8
Documents restraint removal	Always	25	32.9
(from previous group answering	Sometimes	37	48.7
always or sometimes n = 76)	No	13	17.1
	Never finished restraints	1	1.3
Needs improved registry	Yes	119	49.4
	No	122	50.6

**Table 8:** Use of institutional training resources related to physical restraints (n = 241).

Training Resource	Response	n	%
Training module on restraint use	Yes	26	10.8
	No	215	89.2
Training module on restraint prevention	Yes	22	84.6
(over a n = 26)	No	4	15.4
Training module on legal framework	Yes	18	69.2
(over a n = 26)	No	8	30.8

## Data Availability

The data presented in this study are available on request from the corresponding author. The data are not publicly available due to privacy and ethical restrictions related to human participant data and compliance with the General Data Protection Regulation (GDPR).

## Supplementary Materials

The following supporting information can be downloaded at: https://www.mdpi.com/article/10.3390/healthcare14050694/s1, Table S1: Association between perceived sufficient training in physical restraint management and professional characteristics; Table S2: Comparison between psychiatry units and other hospital units; Table S3: Factors associated with perceived need for an additional HCIS restraint registry tool; Table S4: Association between knowledge of physical restraint indications and professional characteristics.

## Abbreviations

The following abbreviations are used in this manuscript:

AEN	Spanish Association of Neuropsychiatry
CISEMadrid	Incident Reporting System of the Madrid Health Service
EHR	Electronic Health Record
HCIS	Healthcare Clinical Information System
PRUQ	Perception of Restraint Use Questionnaire
QR	Quick Response code
SERMAS	Madrid Health Service (Servicio Madrileño de Salud)
TCAE	Nursing Care Assistant (Técnico en Cuidados Auxiliares de Enfermería)
WHO	World Health Organization
