# Supplementary material for: Physical Restraint Use in Acute Care Hospitals: A Diagnostic Study on Knowledge, Documentation, and Patient Safety from a Humanization Perspective
Source: Healthcare (Basel). 2026 Mar 9;14(5):694. doi: 10.3390/healthcare14050694 (PMC12984303; doi:10.3390/healthcare14050694)
Supplement: Supplementary file 1 [file healthcare-14-00694-s001.zip › healthcare-4150820-supplementary.pdf]

Table S1. Association between perceived sufficient training in physical restraint management and professional characteristics

Table 1: Table S1. Association between perceived sufficient training in physical restraint management and professional characteristics

Variable	Sufficient Training n (%)	Insufficient Training n (%)	p-value
Female sex	54 (81.8)	159 (90.9)	0.092
Age group			0.031
Professional category (Nurse)	29 (43.9)	118 (67.4)	<0.001
Psychiatric unit experience			<0.001
Received restraint training	52 (78.8)	57 (32.6)	<0.001
Knowledge of immobilization protocol	39 (59.1)	37 (21.1)	<0.001
Awareness of HCIS registry	47 (71.2)	82 (46.9)	<0.001
Completion of HCIS registry	24 (36.4)	16 (9.1)	<0.001
Knowledge of legal framework	44 (66.7)	38 (21.7)	<0.001

Table S2. Comparison between psychiatry units and other hospital units

Table 2: Table S2. Comparison between psychiatry units and other hospital units

Variable	Psychiatry n (%)	Other Units n (%)	p-value
Received restraint training	15 (75)	94 (42.5)	0.005
Knowledge of immobilization protocol	10 (50)	66 (29.9)	0.063
Awareness of HCIS registry	15 (75)	114 (51.6)	0.044
Knowledge of legal framework	14 (70)	68 (30.8)	<0.001
Need for additional HCIS registry	20 (100)	99 (44.8)	<0.001

Table S3. Factors associated with perceived need for an additional HCIS restraint registry tool

Table 3: Table S3. Factors associated with perceived need for an additional HCIS restraint registry tool

Variable	Additional Tool Needed n (%)	Not Needed n (%)	p-value
Received restraint training	56 (47.1)	53 (43.4)	0.573
Knowledge of immobilization protocol	37 (31.1)	39 (32.0)	0.884
Awareness of HCIS registry	54 (45.4)	75 (61.5)	0.012
Documentation doubts	8 (6.7)	2 (1.6)	<0.001
Ease of registry completion	12 (10.1)	1 (0.8)	0.039
Professional category (Nurse)	60 (50.4)	87 (71.3)	<0.001

Table S4. Association between knowledge of physical restraint indications and professional characteristics

Table 4: Table S4. Association between knowledge of physical restraint indications and professional characteristics

Variable	Knowledge Present n (%)	Knowledge Absent n (%)	p-value
Received restraint training	107 (51.4)	2 (6.1)	<0.001
Knowledge of institutional protocol	73 (35.1)	3 (9.1)	0.003
Awareness of HCIS registry	116 (55.8)	13 (39.4)	0.080
Registry completion	38 (18.3)	2 (6.1)	0.071
Documentation of removal	24 (11.5)	1 (3.0)	0.015
Knowledge of legal framework	81 (38.9)	1 (3.0)	<0.001
